# Client satisfaction and contributing factors towards sexual and reproductive health services delivery system among youth at Family Guidance Association of north Ethiopia (FGAE) clinics, 2023: mixed method study

**DOI:** 10.1186/s12913-024-10874-8

**Published:** 2024-04-16

**Authors:** Niguss Cherie, Yawkal Tsega, Anissa Mohammed, Zinet Abegaz, Abel Endawkie, Yeshimebet Ali Dawed, Natnael Kebede

**Affiliations:** 1 Department of Reproductive and Family Health, School of Public Health, College of Medicine and Health Sciences, Wollo University, Dessie City, Ethiopia; 2 Department of Health System and Management, School of Public Health, College of Medicine and Health Sciences, Wollo University, Dessie City, Ethiopia; 3 Department of Epidemiology and Biostatistics, School of Public Health, Colleges of Medicine and Health Science, Wollo University, Dessie City, Ethiopia; 4 Department of Public Health Nutrition, School of Public Health, College of Medicine and Health Sciences, Wollo University, Dessie, Ethiopia; 5 Department of Health Promotion, School of Public Health, College of Medicine and Health Sciences, Wollo University, Dessie City, Ethiopia

**Keywords:** Client satisfaction, Contributing factors, Sexual and Reproductive Health Services, Ethiopia

## Abstract

**Background:**

The Family Guidance Association of Ethiopia (FGAE) operates as a non-governmental organization dedicated to offering family planning and reproductive health services to the Ethiopian population. The gap in the study regarding client satisfaction and contributing factors towards sexual and reproductive health services for youth at FGAE clinics highlights the need for a comprehensive investigation to fill this void. By conducting a mixed-method study, this research aims to provide a holistic understanding of the factors influencing client satisfaction in the delivery of sexual and reproductive health services to youth at FGAE clinics. The added value of this study lies in its potential to offer valuable insights and recommendations for improving service delivery systems and enhancing client satisfaction levels, ultimately contributing to the overall well-being and health outcomes of youth in North Ethiopia. Therefore study aimed to asses Client Satisfaction and Contributing Factors towards in sexual and reproductive health services delivery system among youth at Family Guidance Association of North Ethiopia (FGAE) Clinics, 2023.

**Method:**

The study was conducted within the clinics of the Family Guidance Association of Ethiopia (FGAE), spanning Dessie, Kombolcha, and Woldia city administrations, involving a participant cohort of 416 clients. Facility-based concurrent type mixed method study design both quantitative and qualitative techniques were applied. Quantitative research employed a simple random sampling technique and conversely, the qualitative study utilized a heterogeneous type of purposive sampling strategy to recruit participants The collected data underwent a rigorous process of entry, cleaning, and coding using Epi-Data 4.6 software, followed by analysis in STATA V17. Descriptive statistics and binary logistic regression were employed to highlight the impact of independent variables on the dependent variable. A more comprehensive examination was provided through multivariable logistic regression. Crude and adjusted odds ratios, along with a 95% confidence interval, were computed, with significance set at a p-value ≤ 0.05.

**Result:**

Nearly more than half of the clients 194 (47.8%) came to receive family planning services followed by maternal and child health 107 (26.4%). Sixty patients (14.8%) didn’t receive all the services they wanted or came for. Half of the participants 30 (50%) raised the unavailability of the service as a reason for not taking the service followed by not having enough time in the clinic 12 (20%). About 65.52% (60.74-70.00%) of the participants were satisfied with the Sexual and Reproductive Health services provided by the clinics of FGAE in northeast Ethiopia. Clients in the age group of 25–34 (AOR = 2.04; 95%CI: 1.11–3.72). Clients who had primary and secondary education (AOR = 2.49; 95%CI: 1.03–6.02) and (AOR = 3.05; 95%CI: 1.25–7.49) respectively. Clients who responded that physicians show respect (AOR = 5.59; 95%CI: 1.89–16.49). clients who received an explanation about the side effects of the utilized methods and follow-up dates (AOR = 4.59;95%CI:1.68–12.53) and (AOR = 2.89;95%CI:1.53–5.49) respectively.

**Conclusion:**

The proportion of client satisfaction with Client Satisfaction in the Services delivery system at Family Guidance Association of Ethiopia (FGAE) Clinics was low as compared to the previous study. Age group 25–34 years, primary and secondary education, showing respect, explaining side-effects and follow-up visits were significant associated factors of client service satisfaction. Enhancing service delivery at Family Guidance Association of Ethiopia (FGAE) Clinics by targeting specific areas identified in the study. Strategies should focus on improving communication regarding side effects, ensuring respectful interactions, and prioritizing follow-up visits, particularly for clients aged 25–34 with primary and secondary education backgrounds.

**Supplementary Information:**

The online version contains supplementary material available at 10.1186/s12913-024-10874-8.

## Background

The Family Guidance Association of Ethiopia (FGAE) is a non-governmental, volunteer-based, not-for-profit organization dedicated to providing comprehensive reproductive health services across various regions of Ethiopia. Over the past five decades, FGAE’s initiatives have evolved significantly, transforming from a single-room clinic staffed by one nurse into a network of 46 integrated sexual and reproductive health (SRH) service delivery facilities. This network includes one Maternal and Child Health (MCH) Center, seven higher Model Clinics, 14 Medium Clinics, and 10 Confidential (Sex-workers Friendly) SRH Clinics. Additionally, FGAE operates 15 youth centers, more than 478 franchised clinics, and over 500 Primary Health Facilities (PHFs) throughout the country, effectively covering almost all regions. These endeavors are coordinated and managed by eight Area Offices strategically positioned across the nation [1, 2]. Consumer satisfaction and perceived quality of outpatient health services, and analysis of consumers’ satisfaction factors will provide valuable insights into healthcare services and consumer satisfaction dynamics [3]. Addressing customer satisfaction and user satisfaction in healthcare services remains a critical focus area in both marketing and healthcare research. It enhances the ongoing efforts to enhance customer and user satisfaction in healthcare services through various strategies and interventions [4, 5].

The satisfaction of clients in sexual and reproductive health (SRH) services serves as a pivotal indicator, gauging the level of contentment a client derives from the services provided by healthcare professionals. Consequently, it mirrors the disparity between the anticipated service and the experience, as perceived by the client [6]. The satisfaction of clients is an essential component of quality and hence is expected to improve through better compliance with the service, at the same time, satisfied clients will generate demand in the community and assist in the recruitment of new clients who can use the services [7].

It has been reported in the meta-analysis that, clients’ satisfaction with family planning services in Ethiopia was 56.78% [6]. Despite high client satisfaction with FP services, the 2016 Ethiopian Demographic and Health Survey (EDHS) reported that over a third of women had stopped using FP within a year [8]. To address this issue, the Ethiopian government has launched a health transformation program aimed at increasing FP use to 55% by 2020 and minimizing the unmet need for FP use to 10% but, progress has been slow since mini-EDHS (2019) found the contraceptive rate to be 41% [9].

Client satisfaction is also an essential determinant of service uptake and continuation because satisfied clients are more likely to revisit the service pass on a positive message to others and continue the use of particular family planning methods [6, 10]. It also signals other aspects of quality of care including structural and process issues of quality of care in FP services. It reflects the perception of healthcare consumers on the quality of care on existing health services [11]. To achieve the target, it is obvious that different health institutions and actors’ involvement shall be important. To contribute to this national effort DMC, DCC, KMSRHS, and WMSRHS are also playing a critical role. The gap in the study regarding client satisfaction and contributing factors towards sexual and reproductive health services for youth at FGAE clinics highlights the need for a comprehensive investigation to fill this void. By conducting a mixed-method study, this research aims to provide a holistic understanding of the factors influencing client satisfaction in the delivery of sexual and reproductive health services to youth at FGAE clinics. The added value of this study lies in its potential to offer valuable insights and recommendations for improving service delivery systems and enhancing client satisfaction levels, ultimately contributing to the overall well-being and health outcomes of youth in North Ethiopia. Therefore study aimed to asses Client Satisfaction and Contributing Factors towards in sexual and reproductive health services delivery system among at youth at Family Guidance Association of north Ethiopia (FGAE) Clinics, 2023.

## Methods and material

### Study area and period

The study will be conducted at FGAE SRH clinics found in Dessie, Kombolcha, and Woldia city administrations which are located in the Eastern Amhara region 401 Km, away from Addis Ababa. Dessie lies 401 km North of Addis Ababa. In the population projection for 2021, the entire population of Dessie was 296,536. A total of 60,315 were women of reproductive age group (15 to 49 years old) of which a total of 9,993 were expected to become pregnant in the year. The city has six hospitals, two of which are government and the other four of which are private. There are also 8 public health centers and 2 Non-Governmental Organizations (NGO) health institutions. The study was completed from August– October 2023.

### Study design and population

Facility-based concurrent type mixed method study design both quantitative (cross-sectional study design) and qualitative (descriptive study design) techniques were applied. The source population for the quantitative study were all clients of FGAE and selected clients of sexual and reproductive health service utilizers at medium clinics of FGAE were study populations. The study participants for the qualitative study were all purposively selected health workers who have adequate experience or are adequately involved in the program and youth who receive services from the Family Guidance Association of Ethiopia’s Northeast Area office in the east Amhara region, Ethiopia.

### Inclusion and exclusion criteria

All clients aged 15–49 years who utilized services were included and those clients who were in critical pain during the data collection period were excluded from the study.

### Sample size determination

The sample size for the quantitative study was calculated using the single population proportion formula for the second, third, and fourth objectives and the double population proportion formula was further calculated for the second objective as well. The sample size for the qualitative study was determined based on the level of saturation. Saturation was considered reached when further addition of participants to the study did not yield any new perspectives or information [12]. The intended number of participants for this study was 10 Key Informant Interviews (KII) and 4 Focus Group Discussions (FGD). The overall client satisfaction in Ethiopia among family planning services was 56.78 [3]. Therefore, with a 5% margin of error and a 95% confidence interval, the sample size was calculated to be 378. Adding a 10% non-response rate, the final sample size was determined to be 416. The sample size for factors with a 95% confidence interval for significant variables from previous research could be calculated using open epi software (Table 1).

**Table 1:** Sample size calculation for factors for the study of Client Satisfaction and Contributing Factors in Sexual and Reproductive Health Services at Family Guidance Association of Ethiopia (FGAE) Clinics

SN	Variables	% control exposed	Power	AOR	Control to-case ratio	Sample size	Total Sample size (add 10% NRR)
1	Waiting time < 30 min [24]	2.3	80%	7.8	1	174	191
2	Privacy ensured during procedure [24]	8.6	80%	7.16	1	68	75
3	The room has posters with key messages on FP [24]	18.24	80%	9	1	38	43

Hence, the largest sample size **416** was considered

### Sampling techniques and procedures

The sample size was distributed proportionally for each FGAE clinic based on the monthly client flow. Consequently, according to the northeast branch of FGAE, there were 31,035 clients in DMC, 9,587 in DCC, 8,675 in KMSRHS, and 10,048 in WMSRHS annually. Therefore, the number of client flows per data collection period (10 days) was estimated to be 162, 51, 46, and 157 in DMC, DCC, KMSRH, and WMSRHS, respectively. As a result, clients were recruited systematically by calculating the sampling interval for each clinic. Hence, every 5th client was interviewed after the first client was recruited using a simple random sampling technique. The sampling techniques for the qualitative study were a heterogeneous type of purposive sampling strategy to recruit the participants. The heterogeneous characteristics of participants were maintained by considering sex and educational level.

### Data collection tools and measurement

The questionnaire was adapted from different literature and pre-tested in a similar setting other than the FGAE clinics of DCC, DMC, WMSRHS, and KMSRHS. Client satisfaction was assessed with exit interviews using twelve Likert-scaled question items. Each item had 5 points, with one denoting “1” for strongly disagree, two for “2” disagree, three for “3” not sure, four for “4” agree, and five for “5” strongly agree. Finally, the mean was computed, and by classifying responses as above/equal and below the mean, they were categorized into “Satisfied (coded as 1)” and “Not satisfied (coded as 0),” respectively. The questionnaire contained the socio-demographic factors of the clients, health service organization experience, reproductive health history, provider competence, and attitude of clients towards SRH service. Besides, the service delivery system of SRH at FGAE was assessed by reviewing strategic and action plans, organizational charts, staffing plans, staff lists, staff job descriptions, and evaluation tools, human resource management manual, financial management and administration, and annual implementation reports. Data were collected with face-to-face interviews with clients, health service providers, and managers, and document review.

The independent variables included socio-demographic factors (age, residence, marital status, educational level, religion, ethnicity, occupation), health facility-related factors (frequency of visit, opening time convenience, privacy maintained during counseling and procedure, cleanliness of the clinic, waiting time, and rooms having posters with key messages of family planning), the information given, and provider-related factors (appointments made for follow-up, clinical staff show respect, providers explain how the method works, providers demonstrate how to use the method, providers describe the possible side-effects, providers describe what to do when a problem occurs, and providers describe the possibility of changing the method when there is a complication) and other interpersonal characteristics. The data collection technique for the qualitative study was key informant interviews and focus group discussions.

Key Informant Interviews involved people focusing on a list of issues regarding a topic with which interviewees had first-hand knowledge to explore coping strategies for the identified factors hindering service uptake in the DMC, DCC, Kombolcha, and Woldia Medium SRH Clinics. A total of 10 key informant interviews were conducted in the study area. Four focus group discussions were conducted with clients who received services from the Family Guidance Association of Ethiopia’s Northeast Area office. The setting was selected by the participants and was convenient and suitable for sitting circularly to allow face-to-face interaction and recording with minimal external disturbance. Three facilitators, including the principal investigator and recorder/note-taker, were assigned for each FGD, with clear roles defined before each session. The principal investigator led the discussion, asked all questions specified in the focus group guide, kept the discussion on track, encouraged participation from all attendees, and took short notes and memos.

### Data quality assurance

**T**he study was collected by 6 BSc nurses working at FGAE clinics. The data collection tool was pre-tested on 5% of the total sample individuals at the youth center. In addition, training was provided for data collectors. Supervisors followed and supervised the overall data collection activities. The quality of the qualitative study was checked by trustworthiness based on Lincoln and Guba’s criteria of credibility, dependability, conformability, and transferability [13]. The principal investigator ensured credibility by pretesting the interview guide in a similar context, using probing and multiple data sources, and inviting participants to review the transcription. Phone calls were made to build rapport, and preconceptions were bracketed to minimize bias. Conformability was ensured by recording all participant activities during interviews, preserving audio-taped interviews, and involving qualitative researchers in peer debriefing for data analysis and interpretations. Dependability was achieved through accurate documentation, frequent checks for spelling errors, and including all relevant documents in the final report, ensuring transparency. Transferability was achieved by providing a clear description of the study setting, sample, and data collection procedures and seeking input from qualitative experts through peer debriefing.

### Data-analysis procedures

Data were entered, cleaned, and coded into Epi-Data 4.6 software and then exported to STATA V17 software for analysis. Simple descriptive statistics such as frequencies mean and standard deviations were done. Following bivariable binary logistic regression, variables with p-values less than 0.25 were selected for inclusion in the multivariable logistic regression model. This step aims to identify independent predictors of the outcome variable while controlling for potential confounders a result, a crude and adjusted odds ratio with a 95% confidence interval was calculated. A p-value of less than or equal to 0.05 was considered significant. The Hosmer and Lemeshow test demonstrated a well-fitted model with a p-value of 0.07, indicating satisfactory agreement between observed and predicted values. Additionally, the absence of multicollinearity suggests that the independent variables in the model are not highly correlated, enhancing the reliability of the regression analysis results. For the qualitative, Audio-recorded interviews were transcribed verbatim and translated to English by the principal investigators, and the translated data were further checked for accuracy by another independent reader. The thematic analysis approach was used to analyze the data. The principal investigators read and re-read the transcriptions several times and listened to the audio-taped interview repeatedly to provide a sense of integrity and understand the meaning of the experiences from the participant’s viewpoint. Each meaning unit was labeled with a code representing its content by open coding, and then similar codes were organized into categories. Atlas. ti software version 7 was used to facilitate data analysis. Two independent coders participated in coding. Categories were peer-reviewed and checked by the co-lead author, and final categories and themes were created. Lastly, the report was written based on categories and predefined themes for presenting the discoveries of the study. Quotes were used to highlight each category and show association with each theme. The findings were triangulated with quantitative findings.

## Results

### Socio-demographic characteristics of study participants

Among the 416 eligible participants, 406 clients participated in the study, reflecting a response rate of 97.6%. Of these, the majority were female, constituting 361 individuals (88.9%), while over half, 209 individuals (51.5%), were married. The average age of the clients was 28.1 years (SD: 6.9), with 202 individuals (49.7%) falling within the 25–34 years age group. Additionally, 364 participants (89.6%) hailed from urban areas. Furthermore, 129 individuals (31.8%) had attained a college education or higher, while 112 (27.6%) identified as housewives. (Table 2). Nearly half of the clients, specifically 194 individuals (47.8%), sought family planning services, while maternal and child health services were sought by 1078 clients (26.4%). Sixty patients (14.8%) were unable to access all the services they required or came for. Among them, half of the participants, totaling 30 individuals (50%), cited service unavailability as the primary reason, followed by insufficient time in the clinic, mentioned by 12 individuals (20%).

**Table 2:** Socio-demographic characteristics of clients served in FGAE clinics, Northeast area office, Ethiopia, 2023

Variables	Categories	Frequency (N)	Percent (%)
Age	15–24	116	28.6
	25–34	202	49.7
	> 35	88	21.7
Sex	Male	45	11.1
	Female	361	88.9
Residence	Urban	364	89.6
	Rural	42	10.3
Marital status	Unmarried	197	48.5
	Married	209	51.5
Occupation	Housewife	112	27.6
	Student	50	12.3
	Gov’t employee	68	16.7
	NGO employee	59	14.5
	Merchant	41	10.1
	Daily worker	68	16.9
	Farmer	8	1.9
Educational status	Unable to read and write	44	10.8
	Primary education	116	28.6
	Secondary education	117	28.8
	College and above	129	31.8
For which services you came for today	Family planning	194	47.8
	Prevention and management of STI	34	8.4
	Maternal and newborn care	107	26.4
	Management of gender-based violence	14	3.5
	HIV testing and counseling	67	16.5
Did you get all the services you wanted	Yes	346	85.2
	No	60	14.8

### Facility related factors

Over 60% of the clients, totaling 252 individuals (62.1%), made multiple visits to the clinic. A vast majority, 389 clients (95.8%), found the clinic’s opening hours convenient, while 382 clients (94.1%) were satisfied with its cleanliness. On average, clients waited 33.5 min in the waiting room (SD: 27.8), with the majority (70.2%) waiting for less than 30 min. Appointment dates were provided on follow-up cards for 321 clients (79.1%). Additionally, healthcare providers explained and demonstrated the use of sexual and reproductive health services to 270 clients (68.2%) and 288 clients (71.3%), respectively. (Table 3).

**Table 3:** Facility-related factors and perceived technical competence of FGAE clinics, Northeast area office, Ethiopia, 2023

Variables	Categories	Frequency (N)	Percent (%)
Frequency of visit	1st time	154	37.9
	2nd and more time	252	62.1
Opening time convenience	Yes	389	95.8
	No	17	4.2
Working hour convenience	Yes	389	95.8
	No	17	4.2
Privacy is ensured during the procedure	Yes	389	95.8
	No	17	4.2
Comfortable with the cleanness of the clinic	Yes	382	94.1
	No	24	5.9
Clinical staff showed respect	Yes	366	90.2
	No	40	9.8
The waiting room has messages about SRH	Yes	329	81.0
	No	77	18.9
Waiting time	<=30 min	285	70.2
	> 30	121	29.8
Follow-up card filled with date of appointment	Yes	321	79.1
	No	85	20.9
Provider explained how to use the SRH service utilization	Yes	270	68.2
	No	126	31.8
The provider demonstrates how to use the required service	Yes	288	71.3
	No	118	28.7
Describe the possible side-effects	Yes	280	69.6
	No	126	30.4
Describe what to do when a problem occurs	Yes	280	69.3
	No	126	30.7
The physician describes a follow-up visit	Yes	309	76.3
	No	97	23.7

### Client satisfaction level towards SRH services in FGAE clinics Northeast area

About 65.52% (60.74-70.00%) of the participants were satisfied with the Sexual and Reproductive Health services provided by the clinics of FGAE in the northeast area office. Moreover, 396(97.54%), 387(95.32%), and 393(96.80%) of the respondents reported that the working hours of the clinics were convenient, the compassionate care, and the consultation time with the health care provider was sufficient to fulfill their needs, respectively. However, about 34(8.37%), 35(8.62%), and 121(29.80%) of the participants stated that their privacy was not secure during the consultation, the service payment was not affordable, and did not get the opportunity to choose family planning (FP) methods in the clinics, respectively (Table 4).

**Table 4:** Client satisfaction level in SRH services provided by FGAE clinics, Northeast area office, Ethiopia, 2023

Variable	Satisfaction	
	Yes	No
	Number (%)	Number (%)
The clinic is easy to get	384(94.58)	22(5.42)
Convenient working hours	396(97.54)	10(2.46)
Get quick services as wanted	395(97.29)	11(2.71)
Sufficient consultation time	393(96.80)	13(3.20)
Get CRC service	387(95.32)	19(4.68)
HCPs listen attentively to client’s needs	382(94.09)	24(5.91)
HCPs give freedom to ask any questions	383(94.33)	23(5.67)
Privacy ensured during consultancy time	372(91.63)	34(8.37)
Get information about SRH services provided in the clinic	322(79.31)	84(20.69)
Get easy IEC materials from the clinic	170(41.87)	236(58.13)
Get detailed information on the FP you used	276(67.98)	130(32.02)
Get the opportunity to choose FP services	285(70.20)	121(29.80)
Clean and convenient clinic	369(90.89)	37(9.11)
Affordable payment for services	371(91.38)	35(8.62)
Advice others to use SRH services in this clinic	377(92.86)	29(7.14)

### Client satisfaction level and facility-related factors by types of FGAE clinics

A noteworthy variation was observed among the different clinics. While WMSRHC had the highest percentage of satisfied clients at 83.58%. On the contrary.KMSRHC and WMSRHC stood out with overwhelmingly positive attitudes at 94.8% and 88.1%, respectively. On the other hand, DCC and DMC had a higher percentage of clients with negative attitudes, indicating a potential area for improvement in these clinics. The analysis of facility-related factors revealed positive trends across all clinics. Opening time convenience, working hour convenience, privacy during procedures, and cleanliness of the clinic were reported as satisfactory by a significant majority of clients in each clinic. Notably, WMSRHC consistently scored high in all these factors, indicating a well-rounded positive facility experience (Table 5).

**Table 5:** Client satisfaction level and facility-related factors by types of FGAE Clinics in Northeast, Ethiopia

Variables	Categories	FGAE Clinics			
		DCC	DMC	KMSRHC	WMSRHC
Client satisfaction level	Dissatisfied	18(27.27%)	78(36.28%)	33(56.90%)	11(16.42%)
	Satisfied	48(72.72%)	137(67.72%)	25(43.10%)	56(83.58%)
Attitude toward (FGAE) Clinics’ service	Positive	25 (37.9%)	67 (31.2%)	55 (94.8%)	59 (88.1%)
	Negative	41 (62.1%)	148 (68.8%)	3 (5.2%)	8 (1.9%)
Opening time convenience	Yes	62(93.9%)	206 (95.8%)	56(96.6%)	65(97.0%)
	No	4(6.1%)	9(4.2%)	2(3.4%)	2(3.0%)
Working hour convenience	Yes	64(97.0%)	203 (94.4%)	57(98.3%)	65(97.0%)
	No	2(3.0%)	12(5.6%)	1(1.7%)	2(3.0%)
Privacy is ensured during the procedure	Yes	62(93.9%)	205 (95.3%)	58 (100.0%)	64(95.5%)
	No	4(6.1%)	10(4.7%)	0(0.0%)	3(4.5%)
Comfortable with the cleanness of the clinic	Yes	59 (89.4%)	199 (92.6%)	58 (100.0%)	66(98.5%)
	No	7(10.6%)	16(6.4%)	0(0.0%)	1(1.5%)

The gap concerning client satisfaction in the clinic appears to be primarily attributed to the narrow and overcrowded waiting areas, particularly in services such as Antenatal Care (ANC) and family planning, as highlighted in key informant interviews and discussions with clinic management. However, confidentiality and privacy do not seem to pose significant challenges to the clinics.

A participant of KII said *“I think all the health care providers including the janitors and securities keep the privacy of clients, since the SRH service needs confidentiality. As a client, some of them may be afraid to use the services here, however, we reassure the clients to keep their secrets” (KII, KMSRHC).*

The clinic’s waiting area, particularly in services like Antenatal Care (ANC) and family planning, is identified as a potential gap in client satisfaction due to overcrowding and narrow space, as indicated in interviews with key informants and discussions with clinic management. However, confidentiality and privacy issues do not seem to present significant challenges to the clinics.

A participant of KII said *“I think all the health care providers including the janitors and securities keep the privacy of clients, since the SRH service needs confidentiality. As a client, some of them may be afraid to use the services here, however, we reassure the clients to keep their secrets” (KII, KMSRHC).*

The clinics used different service promotion strategies to the community through demand creation and use of ICC/BCC materials. But to reach a large community there is a need for large-scale promotion through mass media (radio, TV) and social media (facebook, telegram, and TickTok) to reach more young technology-based generation. There is also a need for promotion banners at different areas of the town and town entry doors to all clinics.

Participants of KII said” *We have a demand creation team in the community, but the promotion is not enough to reach the population to advertise the services. We promote the clinic services through demand creation. We have also posted out said on the clinic and other ICC/BCC materials. We have no media promotion. Better also use promotion banners at different areas of the town and town entry Gates. We use Demand creators, meetings, and IEC/BCC, but do not use media like radio, TV, and other social media communications (KII, DCC, DMC, WMSRH, and KMSRHC).*

Another KII participant reported that*” currently, the number of clients is very low, because, we are not making promotions well through radio or on the street with microphone. Some clients said that they do not know about the FGA availability in the town. But after the clients come to the clinic, we provide leaflets. I think the clinic is not accessible to the clients, this is because, after we moved from the privies place at “KUTEBA” to the current place, the client’s flow decreased significantly. I think the main reason for this decrement is changing the site, and not promoting well about the new site” (KII, KMSRHC).*

Client dissatisfaction primarily stems from the absence of new service components, such as C/S delivery and infertility services, along with extended service waiting times. Additionally, the unavailability of essential services like delivery, Postnatal Care (PNC), pharmacy services, and diagnostic tests in the laboratory can diminish client satisfaction. Other discomforts for clients include overcrowded waiting areas, excessive service integration, lack of accessibility features like ramps for disabled persons at the clinic’s entrance, and weak service integration, particularly in immunization services such as growth monitoring and cold chain management issues.

The KII participant said, *“Clients need new service components to attract clients like C/S delivery, infertility services, decrease client overcrowding and service waiting time, relief the crowding at service payment area, child growth monitoring and promotion and improve child health service room with decoration to attract clients”(KII, DMC).* Another KII participant said *“I think, the unavailability of the different services like delivery, PNC, immunization, Pharmacy etc, may reduce the satisfaction of the clients. If these services had been available, customer satisfaction could have been better (KII, KMSRHC).*

Participants of KII said that *“Common reasons to decrease client satisfaction are unavailability of essential drugs, low numbers of health professionals, and low salary of the employees, long service waiting time and overcrowding of clients at payment area, professional do not wear identification badge among clinic staffs, not wear of uniforms among guards and cleaners(DMC, DCC, WMSR*).

The main logistic challenges were market fluctuation, lack of logistics on the market, Performa process and increased cost of drugs and supplies, shortage of family planning choices (LAFP, COC), condoms, HIV test kits, and STI management kits, vaccine fridge, and cervical cancer screening materials.

A participant of KII said “*There is insufficiency of some inputs like HIV kits, clients go back with no services, lack of CBC machine, shortage of reagents and chemistry machine not working. Medical equipment and materials shortage and not availability is the barrier in this clinic to start services based on clients need*” (KII, WMSRHC).

To enable this clinic to provide quality services, it should be funded sufficiently, fulfill necessary logistics, be filled with enough health professionals as per the standards, and Increase health professionals’ satisfaction as well.

A participant of KII reported that” *The clinic mainly delivers SRH services, but the community demands additional medical and investigation services from the clinic. There is a need for a separate room for radiography and medical services, a need for gynecology and obstetrics specialists, renovation of the clinic building like painting, classroom expansion, improvement staff carrier, need for X-ray machine and service needs attention to improve client satisfaction(KII, DMC).*

Another KII said *“To enable this clinic to provide quality services, it should be funded sufficiently, filled with enough health professionals, Increase health professionals satisfaction, service expansion like delivery, PNC, immunization, Pharmacy etc, may increase the client satisfaction ”KII, WMSRHC, KMSRHC).*

### Attitude towards Family Guidance Association of Ethiopia (FGAE) clinics service

The majority of respondents, accounting for 78.57%, expressed a strongly agree with the belief that clients should utilize health services for SRH. About 73.15% of participants strongly agree that youth should be aware of the importance of SRH services. A gender-related perspective emerged in the data, with 40.15% disagreeing that only females should use health services for SRH. Concerns about the judgmental nature of health providers were evident, with 42.12% disagreeing that health providers exhibit judgmental behavior. Positive responses were observed regarding confidentiality assurance, with 58.13% strongly agreeing that health providers ensure the confidentiality of youth (Table 6).

**Table 6:** Attitude towards family Guidance Association of FGAE clinics, Northeast area office, Ethiopia, 2023

Attitude Assessment	Scale of measurement				
	Strongly agree	Agree	Neutral	Disagree	Strongly disagree
Clients should use health services for SRH for various reasons	319(78.57%)	80(19.70%)	7(1.72%)	0(0%)	0(0%)
Youth should be aware of the importance of SRH service	297(73.15%)	94(23.15%)	12 (2.96%)	2(0.49%)	1(0.25%)
Youths have a harder time getting health services for SRH than adults.	204 (50.25%)	64 (15.76%)	53 (13.05%)	80 (19.70%)	5(1.23%)
Only females should use health services for SRH.	85 (20.94%)	52(12.81%)	63 (15.52%)	163 (40.15%)	43(10.59%)
Health providers are judgmental	112(27.59%)	36(8.87%)	68(16.75%)	171(42.12%)	19(4.68%)
Health providers assure the confidentiality of youth.	236(58.13%)	101(24.88%)	39(9.61%)	27(6.65%)	3(0.74%)
Health workers welcome youth when they come to use SRH service.	270(66.50%)	107(26.35%)	26(6.40%)	1(0.25%)	2(0.49%)

The mean attitude score towards the services provided by the Family Guidance Association of Northeast Ethiopia (FGAE) Clinics was 13.62 ± SD 3.61. Approximately half of the participants, constituting 50.7% (CI: 45.9-55.6%), demonstrated a favorable attitude towards FGAE Clinics, highlighting a positive reception of the services. Conversely, the remaining 49.3% (CI: 44.1-54.1%) exhibited a negative attitude, indicating a discernible proportion of individuals who may have reservations or concerns regarding the services provided by FGAE Clinics.

### Factors associated with client satisfaction in FGAE clinic services

In the binary logistic regression, age, sex, educational status, occupation, residence, frequency of visit, respect is shown for clients, comfort with the cleanness of the clinic, availability of messages in the waiting room about SRH services, demonstration of a method for clients, explaining the side effects of the methods and what to do when a problem occurs, longer waiting times and explaining follow-up time were significantly associated with satisfaction with p-value < 0.25.

In the multivariable logistic regression, only age, 25–34 years, primary and secondary education, showing respect, and explaining side-effects and follow-up visits become important factors for service satisfaction. Clients in the age group of 25–34 were 2.04 times more satisfied than clients in the age group of 15–24 (AOR = 2.04; 95%CI: 1.11–3.72).clients who had primary and secondary education were 2.49 and 3.05 times more satisfied than those clients who were unable to read and write respectively (AOR = 2.49; 95%CI: 1.03–6.02) and (AOR = 3.05; 95%CI: 1.25–7.49) respectively. Clients who responded that physicians show respect were 5.59 times more satisfied than their counterparts (AOR = 5.59; 95%CI: 1.89–16.49). clients who received an explanation about the side effects of the utilized methods and follow-up dates were 4.59 and 2.89 times more satisfied than their counterparts respectively (AOR = 4.59;95%CI:1.68–12.53) and (AOR = 2.89;95%CI:1.53–5.49) respectively (Table 7).

**Table 7:** Factors associated with Client satisfaction of Sexual and Reproductive Health services in FGAE clinics, Northeast area office, Ethiopia, 2023

Variables	Category	client satisfaction		COR (95%CI)	AOR (95%CI)	P-value
		Yes	No			
Age	15–24	70	46	RC	RC	
	25–34	144	58	1.63 (1.01–2.61)	2.04 (1.11–3.72)	0.021*
	>=35	52	36	0.94 (0.54–1.67)	1.59 (0.74–3.38)	0.226
Sex	Male	21	24	0.41 (0.22–0.78)	0.45 (0.19–1.04)	
	Female	245	116	RC	RC	0.062
Residence	Urban	236	128	0.73 (0.36–1.48)	1.08 (0.46–2.57)	0.864
	Rural	30	12	RC	RC	
Occupation	employed	188	106	RC	RC	
	unemployed	78	34	1.29 (0.81–2.06)	1.04 (0.61–1.79)	0.872
Educational status	Unable to read and write	27	17	RC	RC	
	Primary education	85	31	1.73 (0.82–3.59)	2.49 (1.03–6.02)	0.021*
	Secondary education	77	40	1.21 (0.59–2.48)	3.05 (1.25–7.49)	0.009*
	College and above	77	52	0.93 (0.46–1.88)	1.69 (0.71-4.00)	0.184
Frequency of visit	1st time	91	63	RC	RC	
	2nd and more	175	77	1.57 (1.03–2.39)	1.25 (0.72–2.18)	0.439
Comfortable with the cleanness of the clinic	Yes	256	126	2.84 (1.22–6.58)	4.18 (0.94–18.54)	0.060
	No	10	14	RC	RC	
Clinical staff showed respect	Yes	254	112	5.29 (2.59–10.78)	5.59 (1.89–16.49)	0.002*
	No	12	28	RC	RC	
The waiting room has messages about SRH messages	Yes	233	96	3.23 (1.94–5.38)	1.43 (0.72–2.85)	0.300
	No	33	44	RC	RC	
Client waiting time in minutes	< 30 min	192	93	RC	RC	
	>=30 min	74	47	0.76 (0.49–1.19)	0.66 (0.37–1.18)	0.160
Physicians demonstrate how to use	Yes	222	63	6.61 (4.11–10.62)	2.2 (0.85–5.65)	0.101
	No	40	75	RC	RC	
Explain the side-effects	Yes	220	60	6.73 (4.21–10.62)	4.59 (1.68–12.53)	0.003*
	No	43	79	RC	RC	
Problem occurred	Yes	213	67	4.55 (2.89–7.14)	0.57 (0.24–1.34)	0.200
	No	51	73	RC	RC	
Follow-up explained	Yes	229	80	4.77 (2.93–7.75)	2.89 (1.53–5.49)	0.001*
	No	36	60	RC	RC	
Attitude towards FGAE Clinics service	negative	141	59	RC	RC	
	Positive	125	81	0.64 (0.42–0.97)	0.66 (0.39–1.09)	0.119

RC-reference category *Significant at *p* < 0.05 **Significant at *p* < 0.001

## Discussion

According to the findings, the proportion of client satisfaction was 65.52% (95%CI: 60.74-70.00%) in northeast Ethiopia FGAE clinics services. One study in Nigeria found a similar level of client satisfaction with sexual and reproductive health services, with a proportion of 67% among youth accessing services at public health facilities [14]. This finding is lower than a previous study conducted in India [15]. In contrast, a study in Kenya reported a higher proportion of client satisfaction, with 75% of youth expressing satisfaction with sexual and reproductive health services provided by non-governmental organizations (NGOs) [16]. The possible difference may be due to the cultural context in India may have different expectations and standards for client satisfaction compared to the northeast Ethiopia FGAE clinics. Cultural factors such as communication styles, attitudes toward healthcare, and expectations of service quality can influence client satisfaction.

In this study Clients in the age group of 25–34 were more satisfied than clients in the age group of 15–24. In a previous study conducted in India, it was found that clients in the age group of 25–34 were more satisfied with healthcare services compared to clients in the age group of 18–24 [15]. One possible justification for the statement could be that the younger age group (25–34) in this study had access to more advanced and improved healthcare services. This could have resulted in higher satisfaction levels among the younger clients in this study. Additionally, it is possible that the younger age group in this study had different expectations and preferences for healthcare services, which were better met by the healthcare providers, leading to their higher satisfaction levels.

Moreover, the study found that clients who had primary and secondary education were more satisfied than those clients who were unable to read and write. This finding is consistent with previous studies, conducted in India [17], and Ethiopia [18]. One possible justification for this finding could be that clients with higher levels of education may have better health literacy and understanding of their healthcare needs and services. They may also be more empowered to advocate for their health and communicate effectively with healthcare providers. In contrast, a study conducted in Nigeria found no significant association between education level and client satisfaction [19]. The possible discrepancy may be cultural or societal factors may play a role in shaping perceptions of healthcare and satisfaction with services. Further research is needed to explore these potential factors and better understand the relationship between education level and client satisfaction across different contexts.

Furthermore, Clients who responded that physicians show respect were more satisfied than their counterparts. A previous study conducted in the United States also found that clients who perceived their physicians as respectful were more satisfied with their healthcare experience [20]. This suggests that the way physicians interact with their clients can have a significant impact on their satisfaction with healthcare services. Showing respect to clients can help build trust and rapport, which can lead to better communication and more positive healthcare outcomes. Additionally, clients who feel respected may be more likely to adhere to treatment plans and follow-up care recommendations, which can further improve their satisfaction with healthcare services. Therefore, healthcare providers should strive to show respect to their clients as part of their overall approach to providing high-quality care. Finally, clients who received an explanation about the side effects of the utilized methods and follow-up dates were more satisfied than their counterparts. This is similar to the previous studies conducted in Japan [21] and in the United States [22, 23]. One possible justification by explaining the potential side-effects and follow-up dates, physicians are empowering their clients to make informed choices about their treatment and care. When clients are informed about potential side effects and know what to expect in terms of follow-up care, they may feel more in control of their health outcomes and have a better understanding of the treatment process. This, in turn, can lead to higher levels of satisfaction with healthcare services.

### Practical implication of the study

The study on client satisfaction and contributing factors towards sexual and reproductive health services delivery system among youth at Family Guidance Association provides valuable insights that can have profound scientific contributions to the field of public health. By understanding the factors that influence client satisfaction with these services, healthcare providers can tailor their services to better meet the needs of youth, ultimately improving health outcomes and promoting positive behaviours. This research can also inform policy decisions and program development aimed at enhancing the delivery of sexual and reproductive health services to youth, ensuring that they have access to high-quality care that meets their specific needs. Overall, this study has the potential to drive significant advancements in the field of sexual and reproductive health, ultimately benefiting the health and well-being of young people.

## Conclusions

In summary, the proportion of client satisfaction was low in northeast FGAE clinic services. Age group 25–34 years, primary and secondary education, showing respect, explaining side-effects and follow-up visits were significant associated factors of client service satisfaction. Client dissatisfaction arose from a lack of new service components like C/S delivery, insufficient infertility services, and prolonged waiting times. The absence of services such as delivery, PNC, Pharmacy EPI, and a robust laboratory could diminish client satisfaction. Uncomfortable clinic conditions included crowded waiting areas, over-integration of services, accessibility issues for disabled persons, and weak integration of services in immunization, impacting areas like growth monitoring and cold chain management. Enhancing service delivery at Family Guidance Association of Ethiopia (FGAE) Clinics by targeting specific areas identified in the study. Strategies should focus on improving communication regarding side effects, ensuring respectful interactions, and prioritizing follow-up visits, particularly for clients aged 25–34 with primary and secondary education backgrounds.

### Electronic supplementary material

Below is the link to the electronic supplementary material.

Supplementary Material 1

Supplementary Material 2

## Data Availability

No datasets were generated or analysed during the current study.

## Abbreviations

ANC: Anti-Natal Care
BCC: Behavioural Communication Change
C/S: Cease Ran Section
DCC: Dessie Confidential Clinic
DMC: Dessie model Clinic
EPI: Expanded program on Immunization
FGD: Focused Group Discussion
ICC: Information Communication Change
KII: Key Informant Interview
KMSRHC: Kombolcha Medium SRH Clinics
OPD: Out Patient Department
WMSRHC: Woldia Medium SRH Clinics SRH:Sexual and Reproductive Health
PNC: Post-Natal Care
