# Supplementary material for: Client satisfaction and contributing factors towards sexual and reproductive health services delivery system among youth at Family Guidance Association of north Ethiopia (FGAE) clinics, 2023: mixed method study
Source: BMC Health Serv Res. 2024 Apr 16;24:473. doi: 10.1186/s12913-024-10874-8 (PMC11020198; doi:10.1186/s12913-024-10874-8)
Supplement: Supplementary file 1 — Supplementary Material 1 [file 12913_2024_10874_MOESM1_ESM.pdf]

1. Annexes

Annex I: Client exit interview questionnaire

Part I: Information Sheet

"Good morning /afternoon. My name is _________________________ and I am working as data collector with FGA and Wollo University, School of public health on the title "**assessment of service delivery and client satisfaction among FGA model clinics in northeast Ethiopia".** We are conducting interviews to better understand the service delivery modalities and client satisfaction. Participation is voluntary and no remuneration is offered. If you do decide to take part, you can refuse to answer any questions and may stop the interview at any time. All information collected remains confidential and no names are collected. Do you accept to participate?"

Part II – Certificate of consent

With due understanding of the aforementioned information, are you willing to participate in the study? Yes, I consent voluntarily to participate in this study and understand that I have the right to withdraw from the interview at any time without in anyway affecting my right.

District name:	1. Dessie 2. Kombolcha 3. Woldia	
Facility name:	1. DMC 2. DCC 3. KMSRHC 4. WMSRH	
**Part 1: Socio-demographic characteristics**		
**Sr. No**	**Variables**	**Response**
101	Age in years	……………..
102	Sex	A. Male B. Female
103	Residence	A. Urban B. Rural
104	Marital status	A. Single<br>B. Married<br>C. Divorced<br>D. Widowed<br>E. Separated<br>F. Live together

105	Occupation	A. Housewife<br>B. Student<br>C. Government employee<br>D. NGO employee<br>E. Merchant<br>F. Farmer<br>G. other
105	Educational status	A. no formal education<br>B. primary education<br>C. secondary education<br>D. college and above
**Part 2: Preliminary questionnaire information**		
107	What services you came for today?	1. Family planning<br>2. Prevention and management of STI<br>3. Maternal and new-born care<br>4. Management of gender based-violence<br>**5.** HIV testing and counseling<br>**6.** Others
108	Which services you received?	1. Family planning<br>2. Prevention and management of STI<br>3. Maternal and new-born care<br>4. Management of gender based-violence<br>**5.** HIV testing and counseling<br>**6.** Others
109	Did you get all the services you wanted today	1. Yes 2. no
110	If no, why did you not receive all the services youwanted	a. Cost<br>b. Not available<br>c. I didn't have time

		d. I didn't feel comfortable requesting the service<br>e. Service provider does not have time<br>**f.** Other
**Part 3: Facility related factors**		
111	Frequency of visit	1. 1st visit 2. 2nd and more visit
112	Opening time convenience	1. Yes 2. No
113	Working hour convenience	1. Yes 2. No
114	Privacy ensured during theprocedure	1. Yes 2. No
115	Comfortable with the cleanness of the clinic	1. Yes 2. No
116	Clinical staff showed respect	1. Yes 2. No
117	Waiting room has poster about SRH messages	1. Yes 2. No
118	Client waiting time in minutes	------------------------------
119	Follow-up card filled with date of appointment	1. Yes 2. No
**Perceived technical competence**		
120	Provider explained how to use the SRH service utilization	1. Yes 2. No
121	Demonstrate how to use therequired service	1. Yes 2. No
122	Describe the possible side-effects	1. Yes 2. No
123	Describe what to do when aproblem occurs	1. Yes 2. No
124	Describe follow-up visit	1. Yes 2. No
125	Information regarding complication	1. Yes 2. No
**Part 4: Service delivery system**		

**126**	**Health Facility Set up**	1. Yes	2. No
127	Accessible by public transport (no more than a 20 min walk from nearest public transport station)		
128	Located in an area safe for clients especially women and young people		

129	Opening hours are clearly displayed outside the clinic		
130	Facility clean and generally well maintained with adequate ventilation and lighting		
131	The facility has reliable source of power - national grid including back up from a generator or solar system		
132	The facility has adequate personal protective equipment for infection prevention including:		
**Reception and Waiting Area**			
133	There is a registration system that is able to cater for both walk in and appointment clients.		
134	Simple, accurate and up to date information, education and communication (IEC) materials in local and national language		
135	Service Charter is clearly displayed indicating waiting times		
136	Clients have access to water and clean toilets		
137	The waiting room has adequate seating, is properly ventilated and clean		
**Consultation Room**			
138	Room is well ventilated, clean and comfortable		
139	Client's audio and visual privacy is assured with controlled access to visitors and non-essential staff		
140	Appropriate equipment is present/available according to relevant services, and are in use		
141	Functional hand washing facilities (running water, soap and hand towel/drier) are available		
142	Three bin system for segregation of general/non-hazardous waste, infectious waste and sharps is in place		
143	Room has adequate storage space for instruments and needed drugs		
144	The service provider greets clients and treats them with kindness, dignity and respect		

145	The service provider gives the client the choice to meet alone (without partner or care)		
146	Client is provided with information on others SRH services		
147	Provider uses appropriate IEC materials during session (model, charts) and uses language the client understands.		
**Procedure room (LAPM, MVA, NSV, Delivery…etc.)**			
148	SDP has updated guidelines and protocol available and displayed for all services provided including *[ ] LAPM, [ ] MVA, [ ] Delivery [ ] STI / RTI [ ] National HIV testing strategy and algorithm [ ] SGBV [ ] CSE*		
149	Emergency protocols / flow charts are available and displayed		
150	Emergency / resuscitation equipment and supplies are available (check emergency tray)		
151	Clients are provided with a private place for undressing, curtained windows, and cloth or paper drapes to cover the client during the procedure		
**Recovery room**			
152	Recovery room is separate from the waiting areas and easily access from procedure room (same floor and nearby)		
153	Nurses are easily contactable by the client		
154	Is the clinic laboratory well equipped		
**Client Rights and Access**			
155	An alternative payment system is in place for clients who cannot pay (e.g. credit, free or subsidized services)		
156	SDP provides appropriate mobility aids (wheelchairs, crutches, etc.)		
157	SDP uses a range of communication channels and methods to ensure that all people with disabilities and older people, and their careers, have access to health prevention and promotion information, and are equally informed about health services.		
**Part 5: Level of satisfaction of SRH service**			

		1	2	3	4	5
158	I would recommend this health facility to someone else					
159	I would like to come back to this health facility again					
160	Is the provider competent to provide family planning counseling					
161	Provider would not give you anything harmful					
162	Provider could be trusted with a secret					
163	The clinic site is easy to get					
164	Waiting time was not too long					
165	I felt free to ask all questions					
166	Clinic area is clean					
167	I was provided with all the information I needed					
168	When you received counseling the health providers paid more attention toyour privacy					
169	All my health need was met today					
	1. Strongly disagree 2. Disagree 3. Neutral 4. Agree 5. Strongly agree					
	**Part 6: Attitude assessment questions**					

170	Clients should use health service for SRH forvarious reasons	**1.** Strongly agree<br>**2.** Agree<br>**3.** Neutral<br>**4.** Disagree<br>**5.** Strongly disagree
171	Youth should be aware of the importance of SRH service	**1.** Strongly agree<br>**2.** Agree<br>**3.** Neutral<br>**4.** Disagree<br>**5.** Strongly disagree
172	Youths have harder time to get health service for SRH than adults.	**1.** Strongly agree<br>**2.** Agree<br>**3.** Neutral<br>**4.** Disagree<br>**5.** Strongly disagree

173	Only female should use health service for SRH.	**1.** Strongly agree<br>**2.** Agree<br>**3.** Neutral<br>**4.** Disagree<br>**5.** Strongly disagree
174	Health providers are judgmental	**1.** Strongly agree<br>**2.** Agree<br>**3.** Neutral<br>**4.** Disagree<br>**5.** Strongly disagree
175	Health providers assure confidentiality of youth.	**1.** Strongly agree<br>**2.** Agree<br>**3.** Neutral<br>**4.** Disagree<br>**5.** Strongly disagree
176	Health workers welcomed of youth when they come to use SRH service.	**1.** Strongly agree<br>**2.** Agree<br>**3.** Neutral<br>**4.** Disagree<br>**5.** Strongly disagree
177	Are you willing to use SRH services	1. Yes 2.No
