# Supplementary material for: Client satisfaction and contributing factors towards sexual and reproductive health services delivery system among youth at Family Guidance Association of north Ethiopia (FGAE) clinics, 2023: mixed method study
Source: BMC Health Serv Res. 2024 Apr 16;24:473. doi: 10.1186/s12913-024-10874-8 (PMC11020198; doi:10.1186/s12913-024-10874-8)
Supplement: Supplementary file 2 — Supplementary Material 2 [file 12913_2024_10874_MOESM2_ESM.pdf]

Annex II: Key Informant Interviews (KII) guide for service provider

Part I: Information Sheet

"Good morning /afternoon. My name is _________________________ and I am working as data collector with FGA and Wollo University on the title "**assessment of service delivery and client satisfaction among FGA model clinics in northeast Ethiopia".** We are conducting interviews to better understand the service delivery modalities and client satisfaction. Participation is voluntary and no remuneration is offered. If you do decide to take part, you can refuse to answer any questions and may stop the interview at any time. All information collected remains confidential and no names are collected. Do you accept to participate?"

Part II – Certificate of consent

With due understanding of the aforementioned information, are you willing to participate in the study? Yes, I consent voluntarily to participate in this study and understand that I have the right to withdraw from the interview at any time without in anyway affecting my right.

Key Informant Interviews (KII) guiding questions

1. Can you describe your experience with the healthcare providers at the Family Guidance Association of Ethiopia North east area office clinics in terms of their knowledge and expertise in sexual and reproductive health services? How did they address your concerns and provide information and support?

2. How would you rate the set up and logistics at the clinics in terms of accessibility, cleanliness, and privacy? Did you encounter any challenges in accessing the services or maintaining privacy during your visit?

3. What are some of the individual barriers you have encountered when seeking sexual and reproductive health services at the Family Guidance Association of Ethiopia North east area office clinics?

4. Can you describe any specific challenges you have faced in accessing contraception, STI testing, or other sexual and reproductive health services at the clinics? What improvements do you think could be made to address these challenges?

5. Can you share any specific instances where you felt your needs were not adequately met or where you experienced dissatisfaction with the services provided at the clinics? How do you think these instances could have been handled differently to improve your experience?

6. What are the perceptions of policy makers and government officials regarding the delivery of SRH services, and how can policies be improved to better meet the needs of clients?
